# Rapid and Environment‐Friendly LC–MS/MS for Simultaneous Analysis of Amino Acids in Veterinary Medicine

**DOI:** 10.1002/vms3.70212

**Published:** 2025-01-24

**Authors:** HyunYoung Chae, Jae Won Byun, Bok‐Kyung Ku, Ok‐Mi Jeong, Moon Her, TaeWan Kim, JeongWoo Kang

**Affiliations:** ^1^ Animal Disease Diagnosis Division Animal and Plant Quarantine Agency (APQA), Ministry of Agriculture, Food and Rural Affairs Gimcheon‐si Republic of Korea; ^2^ Laboratory of Veterinary Physiology, College of Veterinary Medicine, Kyungpook National University Daegu Republic of Korea; ^3^ Veterinary drugs & Biologics Division Animal and Plant Quarantine Agency (APQA), Ministry of Agriculture, Food and Rural Affairs Gimcheon‐si Republic of Korea

**Keywords:** amino acid analyse, eco‐friendly, green technology, liquid chromatography with tandem mass spectrometry (LC–MS/MS), quality control, veterinary medicine

## Abstract

**Background:**

Amino acid supplements are crucial for animal health and productivity. Traditional analysis methods face limitations like complexity, long testing times and toxic reagents. Therefore, a more efficient and reliable method is needed.

**Objectives:**

This study aimed to develop and validate an efficient method for the simultaneous analysis of eight amino acids commonly used in veterinary medicine: alanine, arginine, glutathione, lysine, ornithine, methionine, threonine and tryptophan.

**Methods:**

We analysed eight veterinary amino acid preparations. From 100 registered products, we selected 35. After confirming ingredients, we diluted them to 1 mg/L with 50% acetonitrile (ACN) and filtered them using a 0.2 µm RC filter for liquid chromatography with tandem mass spectrometry (LC–MS/MS) analysis.

**Results:**

All analytes showed excellent linearity (*r* ^2^ > 0.99) within 0–10 mg/L. The limits of detection (LOD) ranged from 0.04 to 0.83 mg/L, and the limits of quantification (LOQ) ranged from 0.12 to 2.52 mg/L. Average recovery ranged from 92.96% to 105.61%, with relative standard deviations (RSD) from 0.27% to 3.50%, meeting CD 2002/657/EC standards. Six out of the 35 products (17.14%) did not meet regulations.

**Conclusions:**

The method developed in this study offers an efficient and reliable approach for the simultaneous analysis of essential amino acids in veterinary medicine. Implementing this method can improve the quality control of amino acid products, enhancing animal health and productivity. This study also highlights the need for stringent domestic management and continuous monitoring. By overcoming traditional technique limitations, this validated method ensures the quality and efficacy of amino acid supplements in the veterinary industry.

## Graphical abstract

This study developed an eco‐friendly, simple, and rapid method for amino acid analysis, demonstrating its applicability to veterinary amino acid products through a streamlined sample preparation process.

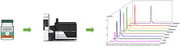

## 1 Introduction

Amino acids are chemically significant compounds in physiology, characterized by the presence of amine and carboxylic acid functional groups. They combine to form proteins and are essential nutrients for the growth and maintenance of physiological functions in animals such as pigs, cattle and poultry. Proteins are composed of around 20 different types of amino acids, categorized into essential and non‐essential amino acids. Essential amino acids must be acquired from external sources to support proper organism function, whereas non‐essential amino acids can be synthesized within the organism (Bosch, Alegría, and Farré 2005; Tome 2004). Among the amino acids, lysine is essential, as it cannot be produced within a pig's body. Lysine serves a crucial function in protein synthesis and muscle development, making it a promising factor for enhancing pig growth (Roy, Lapierre, and Bernier 2000). Methionine is an intracellular antioxidant, and tryptophan improves poultry productivity. In addition, arginine and threonine facilitate the activation of animal immune cells, antibody production and homeostasis (Ognik et al. 2020; Kim et al. 2007; Bhargava et al. 1971).

Several analytical methods are used for amino acid quantification, including high‐performance liquid chromatography (HPLC) (Lamp, Kaltschmitt, and Lüdtke 2018), capillary electrophoresis (Wang et al. 2015), amino acid analysis (Shikha Ojha et al. 2018), gas chromatography (Reddy et al. 2016) and liquid chromatography–mass spectrometry (Li et al. 2017). HPLC is the method most commonly used to analyse non‐volatile, small and polar amino acids (Betts and Russell 2003). Amino acids do not absorb UV light and cannot be identified using UV light. Therefore, a fluorescent substance is attached to an amine or carboxyl group to establish a derivative, which is then analysed using a fluorescence detector (Reddy et al. 2016). The amino acid derivatization reagents include ortho phthalaldehyde (OPA) (Yokoyama et al. 2017), 9‐fluorenylmethoxycarbonyl chloride (FMOC) (Einarsson 1985), ninhydrin (Smon et al. 2019) and phenylisothiocyanate (PITC) (Elkin and Griffith 1985). Derivatization is time‐consuming and may result in incomplete derivatization reactions and reduced stability of the derivatives obtained (Mengerink et al. 2002; Dai et al. 2014). Additionally, existing analytical methods can increase the risk of exposure to hazardous substances in the laboratory environment and experiments due to the use of complex agents and toxic reagents, leading to environmental pollution. Therefore, there is a need to develop analytical methods for measuring amino acid preparations for animals without resorting to derivatization. The liquid chromatography with tandem mass spectrometry (LC–MS/MS) method enables high‐sensitivity analyses, and the method is increasingly being studied; however, most of the studies have focused on food or physiological samples (Gałuszka, Migaszewski, and Namieśnik 2013).

Recent developments in analytical chemistry have led to a notable shift towards reducing or substituting hazardous substances, thereby mitigating environmental and human health risks. Considering the extensive use of various reagents and solvents in such experiments, concerns have arisen regarding their toxicity and environmental impacts. Consequently, there has been an increasing focus on green analytical chemistry (GAC) principles, which were first introduced in 2000 (Armenta et al. 2008; Kaljurand and Koel 2011). Traditional methods for amino acid analysis often rely on substantial quantities of toxic and volatile organic solvents, with insufficient consideration for their potential environmental impact and the safety of laboratory personnel. Consequently, it is imperative to develop novel analytical methods that are less harmful than conventional reagents. Furthermore, there is a pressing need to prioritize the use of non‐hazardous reagents and adopt eco‐friendly approaches to minimize the generation of chemical waste during experiments (Tobiszewski, Mechlinska, and Namie 2010; Pena‐Pereira, Kloskowski, and Namieśnik 2015).

This study presents a methodology that effectively minimizes the use of toxic reagents and organic solvents. The present study reports a novel method of analysing underivatized amino acids using LC–MS/MS with simple pretreatment, short analysis time and high sensitivity and selectivity. Additionally, we aimed to simplify the sample preparation procedure and avoid the use of toxic solvents, such as tetrahydrofuran. We developed a green technology method for analysing amino acids using LC–MS/MS and evaluated its effectiveness in terms of linearity, selectivity, limit of detection (LOD), limit of quantitation (LOQ), accuracy and precision. The developed LC–MS/MS analysis method was employed for monitoring 35 domestically sold products.

## 2 Materials and Methods

### 2.1 Reagents, Solvents and Materials

Amino acid standards (including alanine, arginine, methionine, lysine, ornithine, threonine and tryptophan) were procured from Sigma‐Aldrich (St. Louis, MO, USA). Glutathione, a United States Pharmacopeia (USP) reference standard, was sourced from the Sigma‐Aldrich Chemical Company (Germany). Solvents such as acetonitrile (ACN), methanol (MeOH), sodium phosphate monobasic monohydrate and sodium hydroxide were acquired from Merck (Darmstadt, Germany). Formic acid (98% purity) and phosphoric acid were obtained from Fluka (Ronkonkoma, NY, USA). Distilled water was collected from a Milli‐*Q* Integral System equipped with a 0.22 µm Millipak membrane point‐of‐use cartridge (Millipore, Billerica, MA, USA). All solvents utilized in the analysis were LC–MS grade. A 13‐mm syringe filter (Sartorius, Göttingen, Germany) was employed for sample filtration and was composed of RC material, featuring a pore size of 0.20 µm. Borate buffer (No. 5061‐3339), *o*‐phthalaldehyde (No. 5061‐3335) and FMOC (No. 5061‐3337) were purchased from Agilent Technologies (Basel, Switzerland) for automatic derivatization in HPLC analysis.

### 2.2 Preparation of Standard Stock Solution and Calibration Curve

Alanine, arginine, methionine, lysine, ornithine, threonine and tryptophan standards were prepared at a concentration of 1 mg/mL in distilled water. Glutathione was prepared at a concentration of 1 mg/mL in a 0.5 M hydrochloric acid solution and stored at −20°C.

A working standard mixture of 100 mg/L was prepared by diluting each stock standard solution with 50% ACN to obtain final concentrations of 0, 0.5, 1, 2, 5 and 10 mg/L.

### 2.3 Sample Preparation and Pre‐Treatment Process

This study aimed to analyse eight specific amino acids. Out of the 100 veterinary amino acid formulations approved and registered in the Veterinary Medicine Information System, 35 products were available for purchase. These 35 products did not contain all eight amino acids; rather, each product contained a portion of the target amino acids. After the products with the target amino acids were selected, they were diluted with 50% ACN to reach a final concentration of 1 mg/L. All samples were filtered through a 0.2 µm RC filter before being analysed by LC–MS/MS.

### 2.4 HPLC Analysis

The analysis of eight specific amino acids was conducted using an HPLC system (Agilent 1260 Infinity, Agilent Technologies, Santa Clara, CA, USA) comprising a pump (1260 Quaternary Pump VL, Agilent Technologies), a vial sampler (1260 Vial Sampler, Agilent Technologies) and a fluorescence detector (1260 DAD, Agilent Technologies). A Poroshell 120 HPH‐C18 column (4.6 × 150 mm, 4 µm; Agilent Technologies, California, USA) was employed.

Amino acid derivatization was conducted semi‐automatically in accordance with the instructions outlined by Agilent reagents (Table 1). Detection was carried out using a photodiode array detector at 338 nm. The injection volume for each sample was 10 µL, and the analysis was conducted at a controlled temperature of 40°C.

**TABLE 1:** AUTOSAMPLER PROGRAMMING INSTRUCTIONS.

Line	Function	Amount	Reagent
1	Draw	2.5 µL	Borate buffer
2	Draw	0.5 µL	Sample
3	Mix	3.0 µL in air, max speed, 2 times	
4	Wait	0.5 min	
5	Draw	0 µL	Water (needle wash using uncapped vial)
6	Draw	0.5 µL	OPA
7	Mix	3.5µL in air, max speed, 6 times	
8	Draw	0 µL	Water (needle wash using uncapped vial)
9	Draw	0.5 µL	FMOC
10	Mix	4.0 µL in air, max speed, 6 times	
11	Draw	32 µL	Water (capped vial)
12	Mix	18 µL in air, max speed, 2 times	
13	Inject		

Abbreviations: FMOC, 9‐fluorenylmethoxycarbonyl chloride; OPA, ortho phthalaldehyde.

The mobile phases used were as follows: Mobile phase A consisted of 10 mM sodium phosphate and 10 mM sodium tetraborate (pH 8.2, adjusted with phosphoric acid) in a 50/50 (v/v) ratio, whereas mobile phase B consisted of a mixture of ACN /MeOH /water (45/45/10, v/v/v). Target compound identification relied on the retention times and UV–Vis spectral characteristics of the derivatives generated using corresponding standards. The separation was performed using a gradient programme initially set at 2% eluent B for 1.9 min, followed by a gradual increase in eluent B. The gradient reached 53% at 18.1 min and peaked at 80% at 18.6 min. Eluent B at 80% was maintained for 22.3 min, and at 22.4 min, eluent B was returned to its initial state, followed by equilibration for up to 26 min, resulting in a total analysis time of 26 min.

### 2.5 LC–MS/MS Analysis

The LC–MS/MS analysis was conducted using a Nexera HPLC system (Shimadzu, Kyoto, Japan) coupled with an LCMS‐8045 (Shimadzu), featuring an electrospray ionization ion source operating in multiple reaction monitoring (MRM) mode. In positive mode, the ion source was operated with the following optimized interface parameters: nebulizer gas flow rate of 3.0 L/min, heating gas flow rate of 10.0 L/min, interface temperature of 300°C, desolvation line (DL) temperature of 250°C, heating block temperature of 400°C and drying gas flow rate of 10.0 L/min.

**FIGURE 1:**
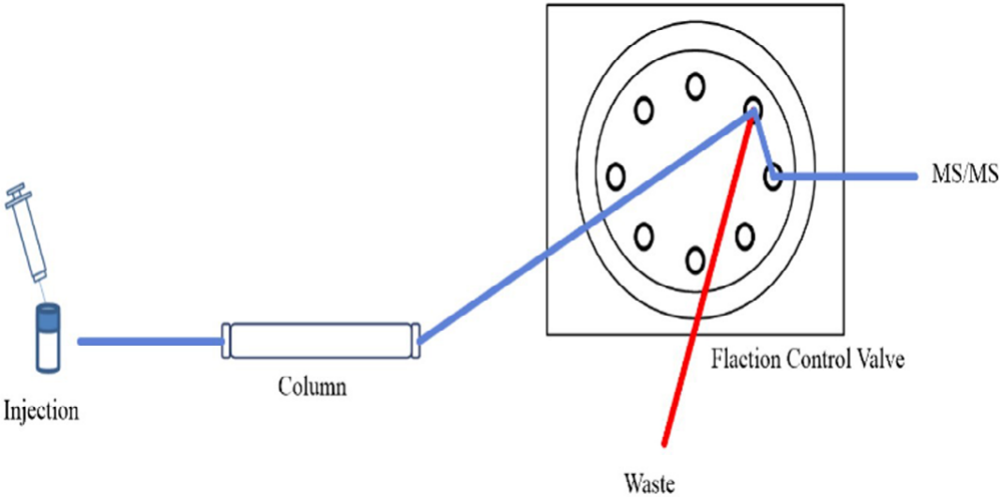
Fractional flow control valve; a fractional flow control valve is installed between LC and MS/MS to minimize MS/MS contamination that can occur due to the influence of various excipients in the product during monitoring.

Chromatographic separation was performed at 40°C using a GIST‐PACK C18 column (Shimadzu, Kyoto, Japan). The flow rate was set to 0.4 mL/min, and the injection volume was 0.5 µL. The mobile phase consisted of two components: (A) 0.1% formic acid in distilled water and (B) 0.1% formic acid in ACN. The gradient programme was as follows: 0–0.5 min, 5% mobile phase B; 0.5–3.0 min, linear increase to 15% B; 3.0–3.5 min, holding at 15% B; 3.5–4.0 min, linear increase to 95% B, followed by a final hold for 0.5 min. The gradient was then returned to its initial conditions within 0.01 min, and the column was allowed to equilibrate for 1.0 min before the next injection, resulting in a total run time of 5.0 min. MRM was utilized to quantify amino acids. A fractional flow control valve was employed to direct the flow to the waste line (except during peak detection) to minimize potential mass spectrometry contamination (Figure 1).

### 2.6 LC–MS/MS Method Validation

The developed method underwent validation according to the guidelines delineated in Commission Decision 2002/657/EC (Kaljurand and Koel 2011). The validation process encompassed various key parameters, including linearity, accuracy, precision, LOD, LOQ, selectivity and inter‐laboratory precision.

Calculations for LOD and LOQ were performed using the equations LOD = 3.3 × (intercept/slope) and LOQ = 10 × (intercept/slope), respectively. Here, the intercept represents the standard deviation of the *y*‐intercept, and the slope is the result of the regression analysis. The first transition was used for quantification, and the other transitions were used for confirmation. Results are expressed as amino acid concentrations in mg/L (*n* = 6). Calibration curves were plotted relative to the nominal concentrations using 1/*x* ^2^ weighting to assess linearity. The concentrations of the six standard solutions, ranging from 0 to 10 mg/L in 50% ACN, were analysed to confirm the linearity and calculate the correlation coefficient of the calibration curve. A recovery experiment was conducted by supplementing an untreated sample with a standard solution to evaluate the accuracy and precision of the analytical method. Recovery was assessed by preparing six replicates of quality control solutions at concentrations of 1 (low), 2 (middle) and 5 (high) mg/L to calculate the accuracy and relative standard deviation (RSD). The inter‐laboratory reproducibility of the developed analysis method was confirmed after verifying the accuracy and precision within the laboratory. The analysis was conducted and verified at Shimadzu Korea's open laboratory under conditions similar to those of the test method.

## 3 Results

### 3.1 HPLC Analysis

**FIGURE 2:**
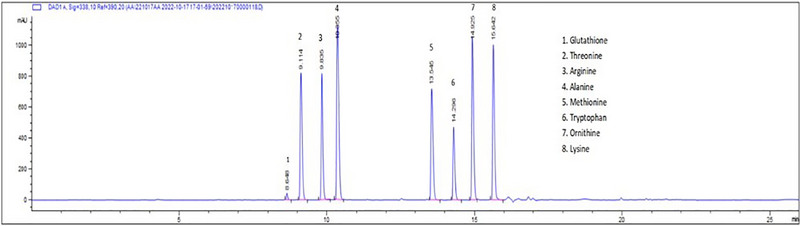
Amino acid chromatogram using HPLC‐UV at 100 mg/L.

HPLC‐DAD was used to screen and confirm the presence of eight compounds in veterinary medicine. The chromatographic conditions and HPLC parameters, including the mobile phase and detection wavelength, were optimized through comprehensive experimentation to assess various factors influencing amino acid analysis. These optimized parameters yielded optimal separation and peak shape. Furthermore, the optimization of chromatographic separation for the target compounds involved adjustments to the retention time of each specific compound. The HPLC chromatograms of the eight compounds are shown in Figure 2. High‐sensitivity analysis was achieved by employing an organic mobile phase characterized by lower absorbance and a suitable buffer solution, effectively reducing noise and minimizing ghost peaks in reversed‐phase chromatography with UV detection. Additionally, combining aqueous and organic solvents resulted in enhanced elution capacity (Abdu Hussen 2022). Successful separation of the target compounds was accomplished by employing a C18 column in conjunction with a mobile phase comprising 10 mM sodium phosphate and 10 mM sodium tetraborate.

### 3.2 LC–MS/MS Analysis

The present study also involved the development of an LC–MS/MS method for simultaneously identifying eight compounds in veterinary medicine. The LC parameters were adjusted, and the chromatographic separation was optimized on the basis of a previous study (Li et al. 2017). The intensity of the MS signal is influenced by several factors, including the choice of organic solvent in the mobile phase. Organic solvents play a crucial role in converting analytes into charged forms, thereby increasing signal intensity. Experiments were conducted under positive and negative polarities, supplementing with H+ or H− to enhance ionization efficiency. The optimal results were consistently achieved when operating in the positive ion mode with the application of formic acid for all the compounds. A comprehensive overview of the LC–MS/MS parameters, including the most prominent monitored transitions for each amino acid, is provided in Table 2. Our analyses were conducted using LC–MS/MS in the MRM mode to ensure high sensitivity and specificity.

**TABLE 2:** Analysis and polarity mode and MS/MS conditions used for quantitation and confirmation of the amino acids.

		Precursor ion		Product ion					
				Quantitation			Confirmation		
Compound	ESI		Q1 pre bias(V)	*m*/*z*	CE(V)	Q3 pre bias(V)	*m*/*z*	CE(V)	Q3 pre bias(V)
**Alanine**	+	90.1	−20	44.0	−15	−15	45.0	−35	−16
**Arginine**	+	175.0	−13	70.1	−24	−25	60.1	−14	−22
**Glutathione**	+	307.9	−23	179.0	−12	−12	76.1	−25	−27
**Lysine**	+	147.0	−11	84.1	−17	−16	86.1	−13	−15
**Methionine**	+	150.1	−11	56.0	−17	−20	61.0	−22	−22
**Ornithine**	+	133.0	−26	70.1	−17	−25	116.2	−13	−11
**Threonine**	+	120.1	−15	56.0	−15	−20	74.2	−12	−27
**Tryptophan**	+	205.0	−15	188.1	−10	−19	146.1	−17	−15

**FIGURE 3:**
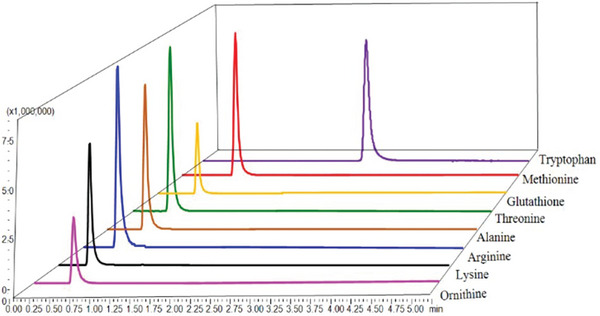
Chromatograms of 8 amino acids mixed standard solutions at 1 mg/L.

Superior chromatographic resolution was obtained using 0.1% formic acid in the aqueous and organic phases on a C18 column. The gradient elution conditions were further optimized through repeated measurements of the standard solution, ultimately achieving satisfactory separation within 5 min. Enhanced peak sensitivity was achieved by employing a fractional flow control valve, which effectively minimized contamination of the tandem mass spectrometer (Figure 3).

### 3.3 Method Validation

A comprehensive method validation was conducted following the guidelines outlined in Commission Decision 2002/657/EC to assess the applicability of the developed method for the quantification of the eight amino acids (European Parliament; the Council of the European Union 2002). The validation encompassed assessing calibration curve linearity, selectivity, accuracy, precision, as well as LOD and LOQ. Linearity was evaluated by generating calibration curves using six concentrations (0, 0.5, 1, 2, 5 and 10 mg/L) of each standard mixture, and excellent correlation coefficients (*r* ^2^ > 0.99) were observed across all analytical ranges. This suggests that an external calibration curve using a standard can reliably serve quantitative purposes.

Selectivity refers to the ability to measure and distinguish analytes when a target compound is mixed with impurities, decomposition products or other compounds (Shah et al. 1992). No interference peaks appeared within ±2.5% in the retention time range; therefore, the developed analysis method had good selectivity.

The determination of LOD and LOQ for each amino acid within the standard mixture was based on the standard deviation of the response and the slope derived from the linearity plot. LOD and LOQ were calculated as 3.3 times the standard deviation of the *y*‐intercept (*σ*) divided by the slope (S) and 10 times σ divided by S, respectively. The resulting LOD and LOQ values ranged from 0.04 to 0.83 mg/L and 0.12 to 2.52 mg/L, respectively, for the eight amino acids. Consequently, all the amino acids analysed in the present study demonstrated suitable sensitivity for applications in the field of veterinary medicine.

Accuracy and precision were determined by assessing the intraday precision. The mixed solution was measured six times per day, and the recovery rate of each amino acid was calculated by comparing the samples (1, 2 and 5 mg/L) for which the standard mixture at each concentration matched the true value as a percentage of the results obtained using our method. Precision was expressed as the RSD derived from the measured standard deviation and mean value. The recovery rates and coefficients of variation fell within the 92.96%–105.61% and 0.27%–3.5% ranges, respectively. All materials exhibited a high recovery rate, despite slight variations observed among the individual substances. Therefore, our proposed experimental method complies with the standard of active ingredient content, falling within the 90%–120% range as stipulated by the ‘Handling Rules of Veterinary Medicinal Products’ (Ministry of Agriculture, Food, and Rural Affairs 2021). Our assay demonstrated an RSD <10% and achieved appropriate LOD and LOQ levels in accordance with the 2002/657/EC guidelines. This provided significant results for amino acid analysis in veterinary medicine (Table 3). Although numerous reports have obtained results using traditional HPLC, the present study showed improved accuracy in amino acid analysis using LC–MS/MS.

**TABLE 3:** Summary of limit of detection (LOD), limit of quantification (LOQ), determination coefficient (*r* ^2^), inter‐lab recovery and relative standard deviations (RSD) of eight amino acids in veterinary medicine.

				Recovery (RSD) (%)			
Analyte	*r* ^2^	LOD (mg/L)	LOQ (mg/L)	Low (1 mg/L)	Middle (2 mg/L)	High (5 mg/L)	Inter‐lab
Alanine	0.99	0.11	0.33	101.96 (0.79)	101.01 (0.27)	97.92 (0.83)	90.00 (0.35)
Arginine	0.99	0.83	2.52	99.43 (2.28)	105.15 (1.07)	98.91 (0.95)	99.30 (0.87)
Glutathione	0.99	0.08	0.27	95.10 (0.91)	97.95 (0.43)	97.95 (0.97)	99.40 (0.54)
Lysine	0.99	0.35	1.07	92.96 (2.13)	99.38 (0.98)	99.85 (0.67)	100.00 (0.85)
Methionine	0.99	0.36	1.11	95.36 (0.32)	97.01 (1.06)	99.39 (0.77)	100.00 (0.71)
Ornithine	0.99	0.44	1.33	103.90 (3.50)	105.61 (2.04)	101.40 (0.40)	100.20 (1.02)
Threonine	0.99	0.09	0.28	102.36 (1.96)	99.58 (0.31)	100.90 (0.28)	99.70 (0.61)
Tryptophan	0.99	0.04	0.12	98.06 (0.77)	98.61 (1.33)	99.34 (0.61)	99.80 (0.79)

### 3.4 Method Comparison

**FIGURE 4:**
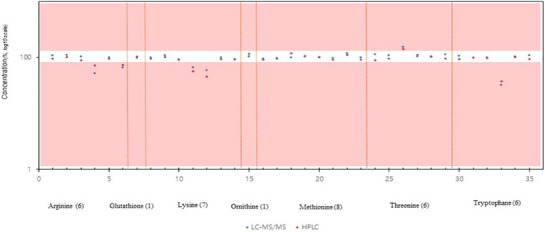
Correlation between HPLC and LC–MS/MS methods for 35 products in domestic distribution. HPLC, high‐performance liquid chromatography; LC–MS/MS, liquid chromatography with tandem mass spectrometry.

Our analytical method represents an advanced version of a widely adopted amino acid analysis technique. We evaluated its performance by comparing it with the existing amino acid analysis method based on LC–MS/MS, which employs analytical equipment distinct from that of the traditional method (HPLC‐DAD). Specifically, we compared the results obtained from a sample solution that could be analysed without derivatization (as is the case with our established method) with those obtained from an existing analytical method that requires derivatization before analysis. Our findings revealed that the LC–MS/MS and conventional analysis methods produced similar quantitative values for the sample product (Figure 4). Traditional amino acid analysis using HPLC typically requires an average analysis time of approximately 20 min. In contrast, our method enables analysis within 5 min per sample. Consequently, our analysis method is more sensitive, accurate and rapid than existing methods.

### 3.5 Evaluation of the Applicability and Validity of Analysis Methods Using LC–MS/MS and HPLC

In this study, we employed both LC–MS/MS and HPLC methods to analyse the amino acid content in veterinary drugs. The results revealed significant variability in amino acid concentrations, with the LC–MS/MS method indicating a range of 32.14%–155% of the declared amount, whereas the HPLC method showed a range of 35.25%–123.14% (Table 4). Among the 35 veterinary products assessed, 29 met the regulatory criteria, adhering to the 90%–120% content range mandated by the Veterinary Drug Handling Regulations. However, six products failed to comply with these prescribed standards.

**TABLE 4:** MONITORING RESULTS OF THE AMINO ACID CONTENT OF 35 PRODUCTS IN DOMESTIC DISTRIBUTION USING HIGH‐PERFORMANCE LIQUID CHROMATOGRAPHY (HPLC) AND LIQUID CHROMATOGRAPHY WITH TANDEM MASS SPECTROMETRY (LC–MS/MS) ANALYSIS METHODS.

Active ingredient	Licensing product	Reference content	HPLC result (%)	LC–MS/MS result (%)
Arginine	A‐1	1.425 mg/mL	94.74	108.74
	A‐2	0.285 mg/mL	101.7	111.79
	A‐3	0.85 mg/mL	90.03	103.97
	A‐4	1.425 mg/mL	72.22	51.42
	A‐5	40 mg/mL	94.56	99.34
	A‐6	125 µg/mL	86.1	66.08
Glutathione	G‐1	360 mg/mL	104.78	97.15
Lysine	L‐1	170 mg/100mL	94.97	99.5
	L‐2	1.02 mg/mL	102.22	108.7
	L‐3	0.525 mg/mL	92.48	92.21
	L‐4	3000 mg/kg	56.07	65.93
	L‐5	20 g/L	35.25	78.45
	L‐6	150 µg/mL	94.15	100.83
	L‐7	34 mg/100mL	92.96	91.04
Ornithine	O‐1	15 mg/mL	117.28	104
Methionine	M‐1	0.525 mg/mL	90.73	95.95
	M‐2	10 mg/mL	95.03	96.83
	M‐3	20 mg/mL	118.12	100.6
	M‐4	3000 mg/kg	106.84	105.07
	M‐5	0.51 mg/mL	101.53	100.05
	M‐6	0.34 mg/mL	92.14	97.57
	M‐7	0.102 mg/mL	112.08	118.53
	M‐8	50 µg/mL	90.8	100.2
Threonine	Th‐1	0.782 mg/mL	90.49	114.9
	Th‐2	0.68 mg/mL	94.7	108.97
	Th‐3	2000 mg/kg	123.14	155
	Th‐4	0.156 mg/mL	110.38	105
	Th‐5	100 µg/mL	103.2	104.25
	Th‐6	0.35 mg/mL	94.62	114.26
Tryptophane	Ty‐1	0.34 mg/mL	92.6	106.69
	Ty‐2	34 mg/100mL	98.82	98.46
	Ty‐3	0.175 mg/mL	97.68	100.43
	Ty‐4	2.5 mg/mL	37.24	32.14
	Ty‐5	0.068 mg/mL	102.41	103.53
	Ty‐6	50 µg/mL	93.68	118.6

Among the amino acids analysed, notable discrepancies were observed in the concentrations of specific amino acids such as arginine and lysine. LC–MS/MS analysis revealed arginine concentrations of 51.42% and 66.08%, compared to 72.22% and 86.10% as determined by HPLC. Similarly, for lysine‐containing products, LC–MS/MS results indicated concentrations of 65.93% and 78.45%, whereas HPLC analysis showed 56.07% and 35.25%. A product containing tryptophan was significantly below the standard, with a content of 32.14% by LC–MS/MS and 37.24% by HPLC. Conversely, a threonine‐containing product exceeded the standard, showing a content of 155.00% by LC–MS/MS and 123.14% by HPLC. The comprehensive analysis of these 35 veterinary products is catalogued in Table 5, with arginine and lysine identified as the amino acids most frequently associated with non‐compliance.

**TABLE 5:** THE PERCENTAGE OF NONCOMPLIANT INGREDIENTS IN DOMESTICALLY DISTRIBUTED AMINO ACID PRODUCT.

Active ingredient	No. of tested samples	Compliance (%)	Non‐compliance (%)
Arginine	6	4 (66.67)	2 (33.33)
Glutathione	1	1 (100)	—
Lysine	7	5 (71.43)	2 (28.57)
Methionine	8	8 (100)	—
Ornithine	1	1 (100)	—
Threonine	6	5 (83.33)	1 (16.67)
Tryptophan	6	5 (83.33)	1 (16.67)
Total	35	29 (82.86)	6 (17.14)

It is important to note that veterinary products on the market may contain various amino acid mixtures; however, not all products include the eight amino acids evaluated in this study. Specifically, alanine was excluded from the analysis as no products containing it were available.

This study underscores the superior efficacy and reliability of the LC–MS/MS method over HPLC for quantifying amino acid content in veterinary drugs. The LC–MS/MS method offers several advantages, including higher sensitivity, greater accuracy and faster analysis times. Traditional amino acid analysis using HPLC typically requires an average analysis time of approximately 20 min per sample. In contrast, our LC–MS/MS method enables analysis within just 5 min per sample, significantly enhancing throughput.

Furthermore, the LC–MS/MS method's ability to analyse samples without the need for derivatization simplifies the workflow and reduces potential sources of error. This increased efficiency and accuracy are crucial for ensuring compliance with established regulatory standards. Precision in analytical methodology facilitates the enforcement of stringent quality control measures in the production and certification of veterinary pharmaceuticals.

Alanine plays an important role in energy metabolism, nitrogen transport, immune function and blood glucose regulation as a non‐essential amino acid, but it can be synthesized within the animal's body. As a result, alanine is considered less critical compared to essential amino acids, and veterinary products typically focus on including essential amino acids (Kim et al. 2007). For this reason, products containing alanine are rare, and it was excluded from the analysis in this study. Therefore, this study focused on essential amino acids such as arginine, lysine and threonine, which are crucial for animal growth and health maintenance.

## 4 Discussion

This study establishes and validates a simple, eco‐friendly, sensitive and rapid quantitative method for the analysis of amino acids in veterinary medicine using LC–MS/MS with a reverse‐phase column. The analysis time is significantly reduced to less than 5 min, compared to other instrumental analyses such as HPLC and gas chromatography. Currently available methods for measuring amino acids present several limitations, including complexity, lengthy sample processing times, extended run times and the use of potentially hazardous reagents such as tetrahydrofuran and ammonium formate for separation. High concentrations of amino acid derivatization reagents can result in the emergence of byproduct peaks and decreased reproducibility, likely due to the concentration of the reagent (Wook Nam et al. 2003). However, the developed LC–MS/MS method allows for rapid and straightforward testing of veterinary medicinal products for impurities and differentiation. Tandem mass spectrometry reduces background noise and increases measurement sensitivity, offering faster and more accurate identification than traditional HPLC methods (Ho et al. 2003).

In addition, this study emphasizes the importance of replacing conventional solvents with less toxic alternatives and minimizing the use of organic solvents to develop eco‐friendly analytical methods. By optimizing particle size and column length, it is possible to enhance analytical efficiency while significantly reducing the amount of organic solvents used, thus minimizing environmental impact. Specifically, the LC–MS/MS method requires only 2 mL of solvent per sample, drastically reducing solvent consumption compared to the 26 mL required for HPLC. This reduction in solvent use helps decrease waste generation and alleviates environmental burdens, contributing to the creation of a more sustainable laboratory environment. Additionally, using shorter columns reduces analysis time and solvent consumption while maintaining comparable analytical efficiency to longer columns with larger particle sizes (Kaljurand and Koel 2011; Chen and Kord 2009). Such eco‐friendly approaches not only protect the environment but also play a crucial role in improving working conditions and safety for personnel in the pharmaceutical industry.

Amino acids classified as veterinary medicines are utilized as feed additives to supplement nutritional deficiencies, prevent diseases, promote growth and improve feed efficiency, as outlined in Article 2, Section 6 of the ‘Handling Rules of Veterinary Medicinal Products’. Currently, the Veterinary Medicine Quality Control Department in South Korea conducts pharmacist monitoring and recall inspections in accordance with veterinary medicine monitoring guidelines. These inspections primarily focus on high‐consumption and noncompliant products. The aim of the present study was to establish a certified, simple and accurate analysis method using LC–MS/MS, and to continuously enhance product monitoring by focusing on amino acids that are relatively difficult to monitor. Verification factors such as linearity, accuracy, precision, selectivity and repeatability were measured to demonstrate the efficacy of the developed method. Additionally, the suitability of the analysis method was confirmed through screening and testing of distributed products. Comparative analysis between the conventional HPLC method, which requires a derivatization step, and the LC–MS/MS method developed for amino acid analysis in veterinary medicine yielded comparable results.

However, in the comparative evaluation of HPLC versus LC–MS/MS for the analysis of arginine and lysine, notable disparities were observed. These differences are attributed to non‐specific interactions during the derivatization phase of HPLC, leading to the misrepresentation of samples containing complex mixtures of components. The derivatization process inherent to HPLC complicates accurate quantification, highlighting a critical limitation of this method. Given these challenges, the demand for an analytical technique that bypasses the derivatization step while maintaining simplicity and efficiency is evident. Our investigation confirms that the LC–MS/MS method excels in selectivity and precision compared to conventional methods, including HPLC. By eliminating the need for derivatization, LC–MS/MS offers a streamlined and more accurate approach to amino acid analysis, proving to be a superior choice for the examination of complex mixtures. This superiority of LC–MS/MS simplifies the analytical workflow and enhances the reliability and accuracy of the results.

Approximately 8 ingredients and 100 products are currently approved according to the Animal and Plant Quarantine Agency (APQA) veterinary medicine information management system. Among them, 35 products were commercially available, with a non‐compliance rate of 17.14%. This study confirms the necessity for stringent content management of amino acid preparations, which are often neglected compared to antibiotics. The analytical method devised in the present study could bolster the management of domestically distributed veterinary amino acid preparations. Previous studies have faced challenges of insufficient quantification when samples are derivatized and analysed using LC–MS/MS (Zytkovicz et al. 2001). The analytical method developed in this study is expected to be instrumental in content management by overcoming existing shortcomings and facilitating accurate quantification.

In conclusion, the processes related to amino acid analysis are expected to become more streamlined and environmentally friendly. We anticipate verifying the amount of amino acids used in veterinary medicine with greater accuracy and detail, achieving higher sensitivity and resolution than HPLC. The present study enhances amino acid analysis techniques in the veterinary sector, enabling more rapid and precise evaluations.

The present study introduces a groundbreaking LC–MS/MS method for rapid and accurate amino acid analysis in veterinary medicine, overcoming the limitations of existing methods such as HPLC. By prioritizing efficiency, eco‐friendliness and safety, the new method substantially reduces analysis time, utilizes less toxic solvents and enhances measurement sensitivity. Furthermore, it provides a more reliable approach for monitoring and managing the quality of veterinary amino acid preparations in South Korea, ensuring compliance with standards. Overall, this advancement facilitates more streamlined, environmentally conscious and precise amino acid analysis processes in the veterinary field. Additionally, this method is expected to have broad applicability, as it can be utilized for the detection and quantification of similar compounds in various analytical fields.

## Author Contributions

Conceptualization: HyunYoung Chae, TaeWan Kim, and JeongWoo Kang. Data curation: HyunYoung Chae, Ok‐Mi Jeong, TaeWan Kim, and JeongWoo Kang. Formal analysis: HyunYoung Chae. Funding acquisition: JeongWoo Kang. Investigation: HyunYoung Chae, Jae Won Byun, TaeWan Kim, and JeongWoo Kang. Methodology: HyunYoung Chae. Project administration: Bok‐Kyung Ku, Ok‐Mi Jeong, Moon Her, and JeongWoo Kang. Resources: Bok‐Kyung Ku and Moon Her. Software: HyunYoung Chae. Supervision: Bok‐Kyung Ku, Ok‐Mi Jeong, and Moon Her. Validation: HyunYoung Chae. Visualization: HyunYoung Chae. Writing–original draft: HyunYoung Chae. Writing–review and editing: HyunYoung Chae, TaeWan Kim, and JeongWoo Kang.

## Conflicts of Interest

The authors declare no conflicts of interest.

### Peer Review

The peer review history for this article is available at https://publons.com/publon/10.1002/vms3.70212.

## Data Availability

The data that support the findings of this study are available from the corresponding author upon reasonable request.
